# A knowledge graph dataset for broiler farming automatically constructed based on a large language model

**DOI:** 10.1016/j.dib.2025.112018

**Published:** 2025-09-09

**Authors:** Nan Ma, Fantao Kong, Jifang Liu, Chenyang Zhang, Chenxv Zhao, Shanshan Cao, Wei Sun

**Affiliations:** a College of Computer and Information Engineering, Xinjiang Agricultural University, Urumqi 830052, China; b Agricultural Information Institute of CAAS, Beijing 100081, China; c Agricultural Economics and Development Institute of CAAS, Beijing 100081, China; d National Nanfan Research Institute of CAAS, Sanya, 572024, China

**Keywords:** Broiler farming, Large language model, Extraction of knowledge, Knowledge graph

## Abstract

With the rapid advancement of artificial intelligence, intelligent farming has become a key trend in modern agriculture. In particular, the application of intelligent systems in broiler farming is essential for enhancing production efficiency and optimizing management practices. Broiler farming is a complex process involving multiple interrelated components. However, existing knowledge graphs primarily focus on disease and prevention, making it difficult to capture the intricate interdependencies within the farming process. This limits the effectiveness of knowledge-based support in decision-making. To develop a high-quality broiler farming knowledge system, this study adopts large language modeling technology to integrate a Chinese corpus and construct a comprehensive knowledge graph dataset covering four core dimensions: broiler breeds, farming environment, feeding management, and disease prevention.

The construction of the dataset involved three key stages. First, text scanning was used to extract information from farming-related literature, while web crawlers collected data from authoritative online sources. The data were then cleaned and manually validated to ensure accuracy and consistency. Second, the DeepKE knowledge extraction framework is used to automatically extract triples related to broiler farming from the text. These are then used as prompts to guide large-scale pre-trained language models (LLMs) to complete and optimize the knowledge, ultimately constructing a relatively complete knowledge graph of broiler farming. Finally, the structured knowledge was stored in a Neo4j graph database to support efficient querying and reasoning.

The dataset not only provides researchers and farms with multidimensional knowledge of the broiler farming domain, but also supports visual management and analysis, enables data-driven inference through large models, and offers new approaches to optimize farming strategies and enhance production efficiency.

Specifications Table

**Table:** 

Subject	Computer Sciences
Specific subject area	Agricultural informatisation
Type of data	Graph Representation and Filtered. Knowledge Graph, backup file.
Data collection	The book data were sourced based on recommendations from domain experts and digitized using CamScanner (https://www.camscanner.com). Website data were obtained using Python-based web scraping from broiler farming websites(For detailed data, see Tables 1 and 2). To ensure data quality, manual cleaning and proofreading were performed. During data collation, duplicate content from different sources was compared, and the most accurate version was retained. Complementary information was merged where applicable to enhance data coverage, improve consistency, and ensure completeness.
Data source location	Country: China
Data accessibility	Repository name: Mendeley Data Data identification number: DOI: 10.17632/ykbn2t62xh.4 Direct URL to data: https://data.mendeley.com/datasets/ykbn2t62xh/4 Instructions for accessing these data: The dataset repository includes a dump file that can be directly imported into Neo4j Desktop, which is available for download at https://neo4j.com/download/. After downloading and installing Neo4j, simply follow the instructions provided at https://neo4j.com/docs/desktop-manual/current/operations/create-from-dump/#::text=Once%20you%20have%20a%20dump,when%20creating%20a%20new%20DBMS. Detailed information about the entities, relationships, and other aspects can be found in the Supplementary Documents.zip.
Related research article	None

## 1 Value of the Data

• This dataset provides a structured and comprehensive knowledge graph of broiler chicken farming, integrating four critical dimensions: breed characteristics, farming environment, feeding management, and disease prevention. It significantly extends the scope of existing broiler knowledge graphs by incorporating multi-stage and multi-dimensional farming knowledge.
• The knowledge graph supports vertical-domain retrieval tools by offering structured semantic data, which enhances retrieval accuracy and reduces query time, thereby improving access to domain-specific information.
• Leveraging its reasoning and analytical capabilities, the dataset can assist in decision-making throughout the broiler production process, promoting precision farming practices. Moreover, it serves as a foundational resource for training domain-specific large language models, contributing to the advancement of intelligent farming systems.

## 2 Background

Global chicken meat production has experienced steady growth, outpacing the growth rates of pork, beef, and lamb over the same period [1]. Broilers, as the predominant form of poultry production worldwide, have accounted for over 85 % of total poultry output since 1961 [2].To meet growing market demand, broiler farming has advanced in scale and specialization[3]. However, challenges remain in achieving intelligent development, including fine-tuning of environmental conditions [4], early disease detection and prevention [5], and implementing precise feeding strategies across different growth stages [6].

Current data in the broiler farming domain are fragmented, heterogeneous, and difficult to mine, which hinders the development of knowledge-driven decision-making and intelligent recommendation systems.Compared to general knowledge graphs, domain-specific graphs offer focused coverage, detailed classification, and richer semantic relationships—making them more suitable for professional applications. Examples include the GeoNames dataset in geography [7,8], ICD-11 and UMLS in medicine [[9], [10], [11]], and the Alibaba Semantic Knowledge Base in e-commerce [12], each of which supports high-quality knowledge systems tailored to their specific domains.

In poultry farming, existing studies have primarily focused on developing disease-related knowledge resources. For instance, researchers have created a Chinese named entity recognition corpus covering 28 poultry diseases, which supports the extraction of symptoms, affected body parts, and medications [13]. Another study employed entity-relation extraction methods to build a poultry disease knowledge graph aimed at improving the expression and reasoning of structured information, such as treatment plans [14]. However, broiler chicken farming is a complex, multi-stage process with tightly integrated components. Disease-focused knowledge graphs often fall short in capturing the full complexity of broiler farming, limiting their effectiveness in supporting comprehensive decision-making across all stages.

This study targets four core dimensions of broiler farming: breed characteristics, rearing environment, feeding management, and disease prevention. Leveraging large language models alongside entity and relationship extraction techniques, this study automatically constructs a structured and reliable knowledge graph for broiler farming. Compared to existing poultry disease corpora and knowledge graphs (see Table 1), the proposed dataset offers broader data types, richer content dimensions, and stronger support for downstream applications.

**Table 1:** Comparison of datasets.

**No.**	Dataset	Domain	Number of Data Types		Dimensions Covered	Supported Tasks	Open Access
1	CDNER Dataset	Poultry Disease (NER Corpus)	5	Type, Disease, Symptom, Body part, Drug	Disease Prevention	Named Entity Recognition	Yes
2	Poultry Knowledge Graph	Poultry Disease (Knowledge Graph)	5	Species, Diseases, Symptoms, Drugs, Treatment Measures	Breed and Disease Prevention	Intelligent Diagnosis	No
3	Broiler Farming Knowledge Graph	Broiler Farming	32	hatching technology, biological characteristics,… , .et al.	Breed, Farming Environment, Management Practices, and Disease Prevention	Question Answering, Vertical Semantic Search	Yes

## 3 Data Description

**Fig. 1:**
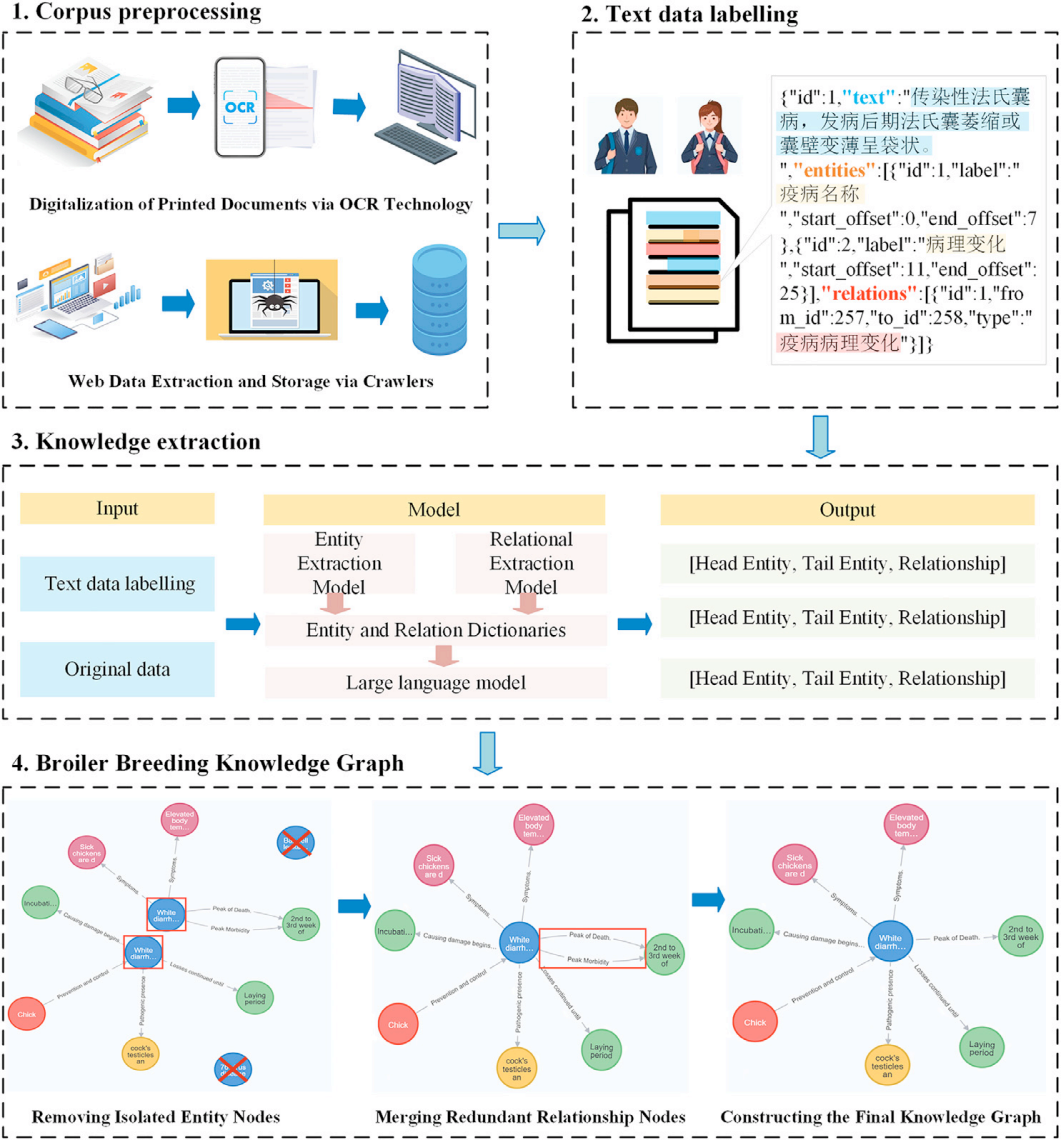
Flowchart of knowledge graph construction.

The construction of this dataset involves four main stages: data preprocessing, manual annotation, knowledge extraction, and knowledge graph storage and visualization, as illustrated in Fig. 1. First, textual data were collected from printed literature and web sources through OCR (Optical Character Recognition) and web crawling technologies, followed by a series of preprocessing steps such as cleaning and structuring. Second, annotated samples were used to train named entity recognition and relation extraction models, enabling the automatic identification of domain-specific entities and relationships. Finally, large language models were employed to assist in knowledge completion and enhancement, and the resulting broiler breeding knowledge graph was stored and visualized using the Neo4j graph database.

The dataset consists of two files with the following structure:

Dataset/

├—— Knowledge Graph /

│ ├—— Broiler_Breeding_neo4j_20,250,519.dump

│ └—— README.md

└—— Supplementary documents/

### 3.1 Data sources

The dataset is derived from 18 Chinese-language books[[15], [16], [17], [18], [19], [20], [21], [22], [23], [24], [25], [26], [27], [28], [29], [30], [31], [32]] and 2522 Chinese web pages[[33], [34], [35], [36], [37]] in the broiler farming domain, providing a comprehensive corpus for subsequent knowledge extraction and graph construction. Book sources were recommended by field experts and converted into digital format using scanning tools. Detailed book information is provided in Table 1. To further expand the dataset’s coverage, broiler farming–related webpages were collected using the Octopus Collector tool (https://www.bazhuayu.com/). All retrieved content was carefully reviewed for topical relevance and source credibility before being compiled into structured document format, as summarized in Table 2.

**Table 2:** Book data and sources.

No.	Title (English Translation)	Editor(s)	Publisher	Time
1	Ecological Broiler Farming	Xu Jianqin, Liu Fenghua	China Agriculture Press	2011
2	Efficient Production Techniques for Moderately Scaled Broiler Farms	Huang Yinyun, Chen Ming	China Agricultural Science and Technology Press	2015
3	Illustrated Guide to Efficient and Healthy Broiler Farming	Li Shuqing, Cao Dingguo	China Agriculture Press	2018
4	Efficient Rearing and Feed Formulation for Broiler Breeders	Qi Xiaolong	China Agriculture Press	2017
5	New Technologies for Modern Broiler Breeder Management	Chen Heqiang, Lian Jinghua	China Agricultural Science and Technology Press	2015
6	How to Improve Profitability in Broiler Farming	Wei Gangcai, Niu Keke	China Machine Press	2021
7	Illustrated Signals and Management in Broiler Farming	Li Lianren	Chemical Industry Press	2015
8	Standardized Management Techniques for Commercial Broiler Farming	Lü Xiangjun, Zhou Side	China Agricultural Science and Technology Press	2012
9	Key Techniques for Production and Operation in Large-Scale Broiler Farms	Wang Haiwei, Wang Zhen	China Agriculture Press	2019
10	Manual for Monitoring and Optimizing Broiler Farming Environments	Zhang Minhong, Zhou Ying	China Agriculture Press	2021
11	Handbook on Production and Business Management of Large-Scale Broiler Farms	Zhang Jing, Ma Jifei	China Agriculture Press	2014
12	Illustrated Guide to Large-Scale White-Feather Broiler Farming	Huang Yankun	Henan Science and Technology Press	2021
13	100 Questions on Healthy Broiler Farming Techniques	Cheng Taiping, Li Peng	China Agriculture Press	2015
14	Poultry Farming and Disease Prevention	Zang Sumin	China Agricultural University Press	2012
15	New Training Manual for Broiler Farm Workers	Li Lianren	China Agricultural Science and Technology Press	2017
16	Broiler Production Guidebook	Xi Keqi	China Agriculture Press	1998
17	Broiler Rearing Manual	Yang Quanming, Diao Youxiang	China Agricultural University Press	2007
18	Promoted Techniques for Standardized Broiler Farming	Li Shuqing, Cao Dingguo	China Agricultural Science and Technology Press	2016

### 3.2 Data labelling

The study labeled 33 entity classes and 338 relationship types across four thematic categories, as shown in Table 3. Initially, book data were manually annotated, followed by expert review for accuracy. This process resulted in the labeling of 4070 entities and 1591 relationships from books, and 8568 entities and 1159 relationships from website data. To enrich the labeled samples, a weakly supervised approach was employed for batch labeling.

**Table 3:** Website data and sources.

No.	Website source	Number of pages	Time
1	https://www.jbzyw.com/	1534	Accessed 30 April 2025
2	https://www.ckexc.com/	706	Accessed 30 April 2025
3	http://www.xinnong.net	135	Accessed 30 April 2025
4	https://yb.caaa.cn/	60	Accessed 30 April 2025
5	http://www.cyangzhi.com	87	Accessed 30 April 2025

The ontology used for entity and relation annotation was developed using a bottom-up, data-driven approach. Specifically, entity types and relation categories were induced from frequently co-occurring patterns in the domain corpus and iteratively refined through manual annotation and domain expert review. This pragmatic design strategy reflects the emergent structure and semantics of real-world text, thereby achieving high contextual relevance and adaptability to the broiler chicken farming domain.

### 3.3 Knowledge graph

The broiler breeding knowledge graph constructed in this study contains 26,811 entities and 18,500 relationships.From the dimension of broiler breed, it contains breed name, breeding system, hatching technology, biological characteristics, internal structure, external structure, and body characteristics. From the dimension of breeding environment, it contains light control, ventilation control, temperature control, relative humidity control, farm location requirements, drinking water management and other entities. From the dimension of feeding management, it contains production performance, growth and development period, feeding standard, feeding management. From the epidemic prevention dimension, it contains epidemic name, pathogen, symptoms, transmission mode, epidemic prevention and control, epidemiology, pathological changes, and drug use requirements. An overview of the knowledge graph is summarized in Table 4.

**Table 4:** List of entity classes, relationship terms (partial).

No.	Head Entity Type	Tail Entity Type	Relationship
1	疫病名称 (Disease Name)	防治 (Prevention and Control)	疫病防治 (Disease Prevention and Control)
2	症状 (Symptom)	诊断 (Diagnosis)	疫病诊断 (Disease Diagnosis)
3	品种名称 (Breed Name)	外部构造 (External Morphology)	品种体型特征 (Breed Physical Characteristics)
4	品种生长阶段 (Growth Stage)	光照控制 (Lighting Control)	生长阶段-光照管理 (Growth Stage–Light Management)
5	喂料管理 (Feeding Management)	饲料添加剂 (Feed Additive)	饲料成分-添加剂 (Feed Component–Additive)

## 4 Experimental Design, Materials and Methods

In this study, Chinese-language text data related to broiler farming were collected from both books and websites. Entity and relationship extraction models were developed through manual annotation and related techniques. A broiler farming knowledge graph was then automatically constructed using natural language processing methods, including large language models, to provide a reliable foundation for research. The graph database illustrates the interactions and relationships among broiler breeds, breeding environments, feeding management, and disease prevention.

### 4.1 Data preprocessing

For the document data collected from different sources, a manual correction process was first carried out, focusing on checking and correcting typos, formatting issues and other errors introduced by scanning or optical character recognition (OCR) that arose from the conversion of physical books into digital format. Subsequently, all data were synthesised, identifying and combining information with the same representation type, while removing duplicates to simplify the dataset and enhance its consistency and usability.

**Fig. 2:**
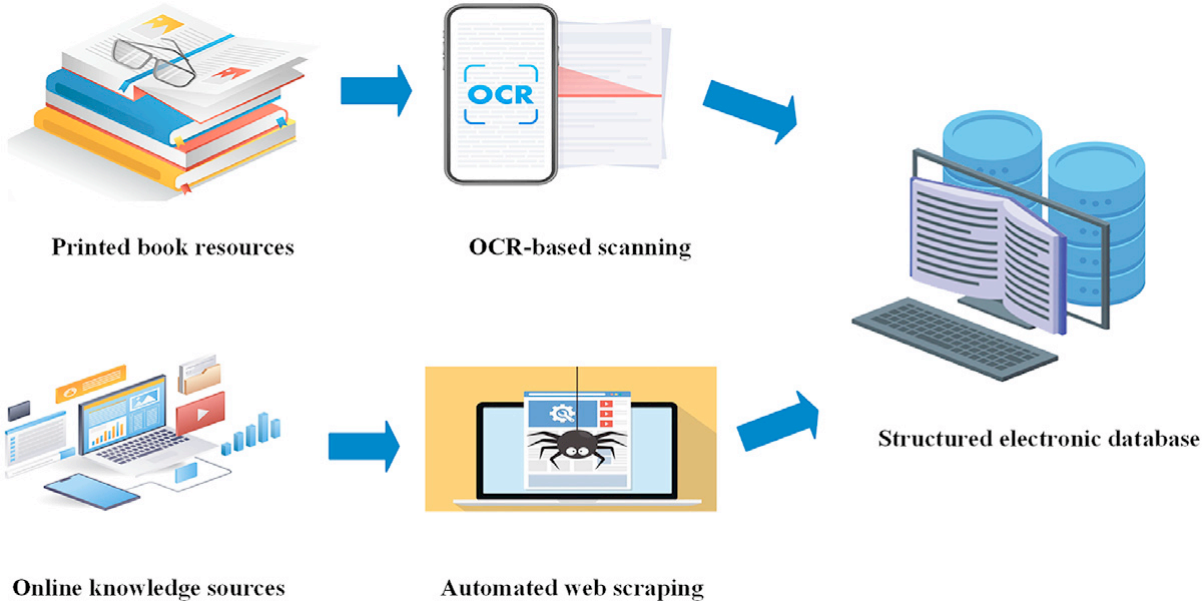
Flowchart of pre-processing of data from different sources.

Common issues included inconsistent spelling (e.g., “broiler feed” vs. “broiler-feed”), mixed punctuation (e.g., English vs. Chinese commas), and vague entity boundaries (e.g., truncating “broiler growth environment temperature” to “broiler growth”). To resolve these problems, a three-stage cleaning pipeline was implemented: (1) automatic preprocessing, using regular expressions and a standardized entity dictionary to unify naming formats and eliminate redundant symbols; (2) manual review to identify semantic overlaps, merge synonymous expressions, and eliminate duplicate triples; (3) expert validation, where livestock and poultry specialists independently assessed the integrated dataset, verifying factual accuracy, biological coherence, and semantic clarity. Finally, high-quality effective data on broiler farming were formed, providing a reliable basis for subsequent applications (Fig. 2).

**Fig. 3:**
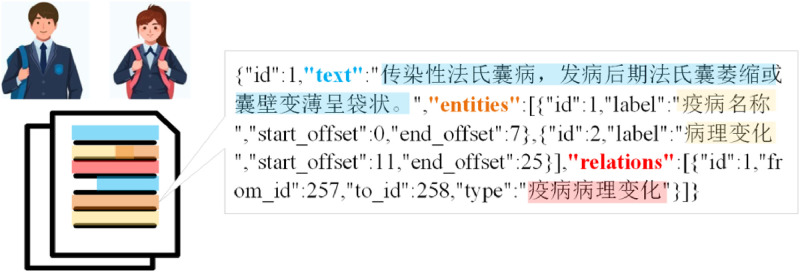
Sample entity and relationship labelling.

To fully leverage AI technologies, entities and their relationships were efficiently extracted from the collected data to build an accurate and comprehensive knowledge graph. Doccano (https://github.com/doccano/doccano) was selected as the primary annotation platform. Firstly, the text data is sliced. Second, the text of each slice is manually annotated according to the entity class and the relational lexicon. Finally, domain experts performed thorough quality control and data validation on the annotated text to ensure the accuracy and consistency of the results. Furthermore, to efficiently expand the annotated dataset, this study employs a weakly supervised annotation strategy to enable batch, semi-automatic extraction of entities and relationships. This hybrid approach greatly enhances the efficiency of triplet extraction (Fig. 3).

To assess annotation consistency, a supplementary inter-annotator agreement (IAA) experiment was conducted. Two domain experts independently annotated 100 randomly selected sentences using the Doccano platform. The calculated Cohen’s Kappa coefficient was 0.81, and the macro-average F1 score between annotators reached 0.80, indicating significant consistency. These results confirm the quality and reliability of the annotated data used for model training.

### 4.2 Knowledge extraction

**Fig. 4:**
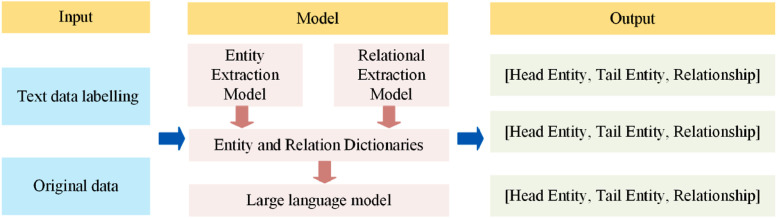
Knowledge extraction process.

DeepKE is an integrated framework that combines Named Entity Recognition (NER), Relation Extraction (RE), and overall knowledge extraction capabilities [38]. However, its performance depends heavily on large volumes of domain-specific annotated data, limiting its scalability across broader corpora. In contrast, Large Language Models (LLMs) can extract knowledge triples without labeled data but are prone to hallucinations, potentially undermining the scientific accuracy of the results. To combine the strengths of both approaches, this study introduces a hybrid DeepKE+GPT pipeline specifically designed for knowledge extraction in broiler farming. First, DeepKE is used to extract entities and relations from structured book data (https://github.co*m/z*junlp/DeepKE), which are then compiled into entity and relation dictionaries. These dictionaries serve as prompts to guide the LLM in automatically extracting knowledge from web-sourced data. The complete extraction workflow is illustrated in Fig. 4, while hardware specifications for model execution are provided in Table 5, Table 6.

**Table 5:** Summary of knowledge graph.

Parameters	Values
Relationships count and types	18,500 (consisting of 31 types)
Label	31 (Types of nodes)
Total nodes	26,811

**Table 6:** Hardware configuration.

Platform	Python, VsCode
systems	Ubantu20.04
GPU	Dell Precision T7960, A6000 graphics card

#### 4.2.1 Entity extraction

**Fig. 5:**
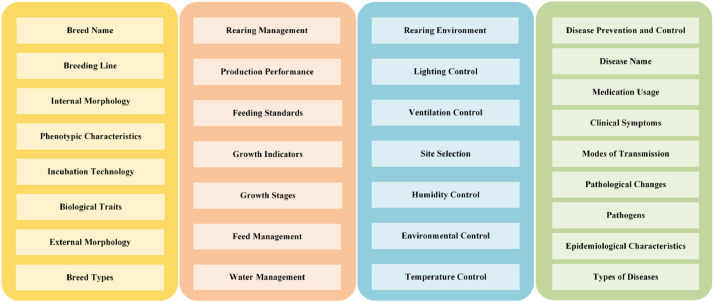
Entity class extracted from knowledge graphs.

First, labeled data are used to identify and organize all entities and their labels, forming a dictionary of broiler farming entities.Second, the dataset is divided into training, testing, and validation sets in an 8:1:1 ratio.Finally, the partitioned dataset is input into an LSTM-CRF model for training, resulting in a broiler farming entity extraction model. The extracted entity categories are presented in Fig. 5.

To quantitatively evaluate the performance of the entity recognition model, the macro-average accuracy, recall, and F1 score of the LSTM-CRF model were calculated, as shown in Table 7. The NER model demonstrates stable overall performance, achieving strong results in recognizing key entities. Notably, entities such as ‘disease name’ and ‘ventilation control’ were recognized with high accuracy, while low-frequency or context-sensitive entities like ‘vaccine’ and ‘feeding management’ showed lower performance. The reason for this is that the low frequency of occurrence makes recognition difficult. In the later stages, data augmentation and few-shot fine-tuning techniques can be explored to improve the recognition performance of such critical but underrepresented categories.

**Table 7:** Training results of the entity extraction model (Partial).

Entity Label	Precision	Recall	F1-Score	Support
B-品种名称(Breed Name)	0.7862	0.8847	0.8326	607
B-疫病名称(Disease Name)	0.919	0.8929	0.9058	915
B-防治 (Prevention/Treatment)	0.9765	0.9525	0.9644	611
I-疫苗(Vaccine)	0.8706	0.6567	0.7487	533
I-喂料管理(Feeding Management)	0.9176	0.6047	0.729	129
I-体型特征(Body Shape)	0.8782	0.7569	0.8131	181
B-通风换气控制 (Ventilation Control)	0.9665	0.9788	0.9726	236
macro_avg	0.9208	0.8726	0.8925	341,360
accuracy	–	–	0.9915	341,360

Due to the highly imbalanced label distribution in the NER task—particularly the dominance of the non-entity label ‘O’—overall metrics such as accuracy may not objectively reflect the model’s actual ability to identify entities. Therefore, macro-averaged Precision, Recall, and F1-score are adopted as the primary evaluation metrics to more comprehensively assess the model’s performance across different entity types.

#### 4.2.2 Relationship extraction

Based on the extracted entity types and annotated relationships, the data are structured into a broiler farming relationship dictionary in the form of ‘head entity–relationship–tail entity’. This dictionary is then split into training, testing, and validation sets using an 8:1:1 ratio. The dataset is fed into the BERT-base-Chinese model for training to develop the broiler farming relationship extraction model.

For the relation extraction task, BERT-based models were evaluated on the same data partition. As shown in Table 8, the model demonstrates excellent performance on the test set, achieving a Macro-F1 score of 0.9520. This indicates that the model can reliably and consistently identify various relationship types. The model also achieves a Precision of 0.9547 and Recall of 0.9520, reflecting strong accuracy and coverage in relationship extraction. An overall accuracy of 95.20 % further indicates that the majority of samples are correctly classified.

**Table 8:** Evaluation metrics of the relationship extraction model.

Metric	Value
Test Macro-F1	0.9520
Test Precision / Recall	0.9547 / 0.9520
Test Accuracy	95.20 %
Test Loss	0.2428

#### 4.2.3 Large language model-based extraction

**Fig. 6:**
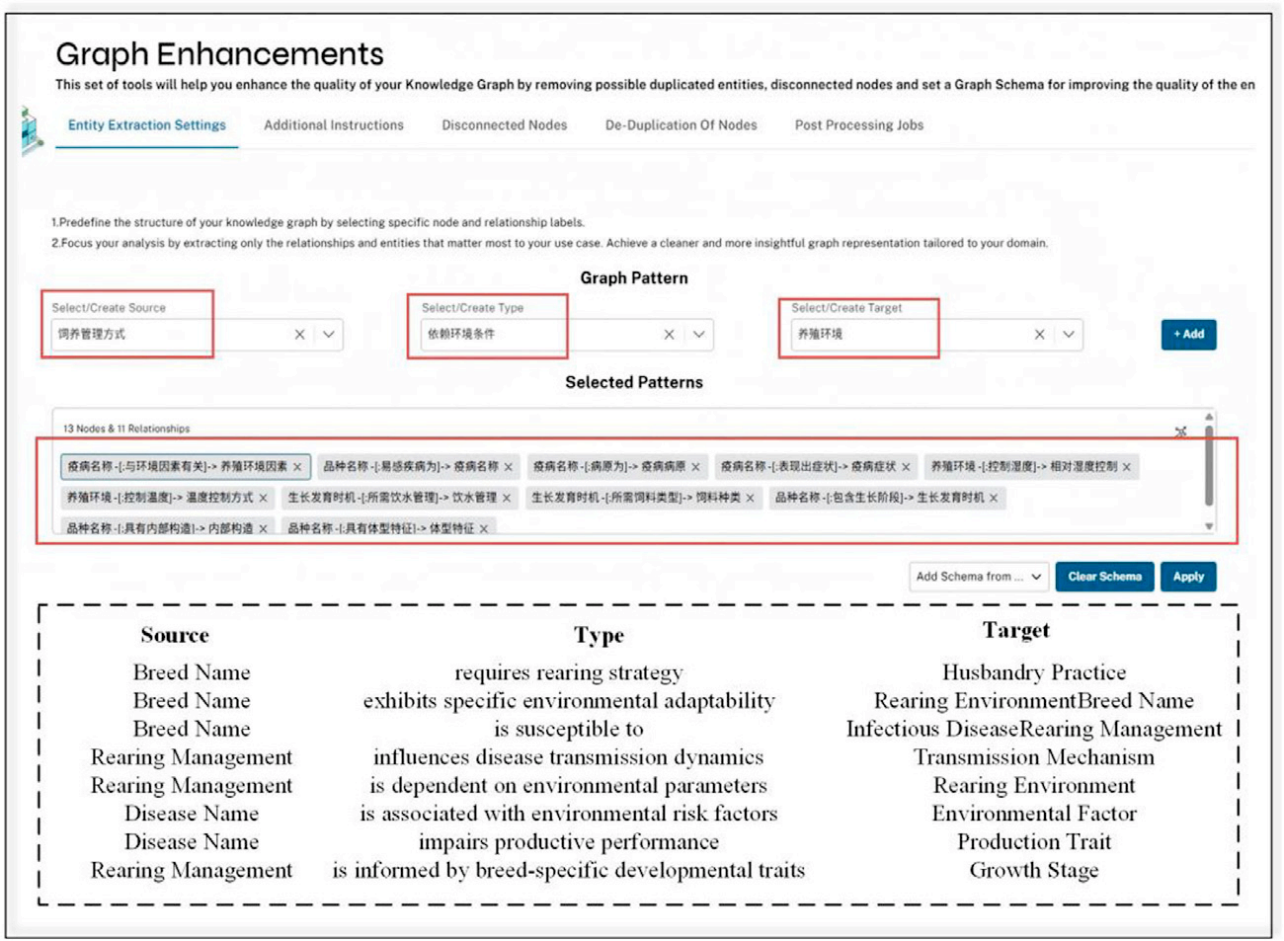
Add domain constraints.

**Fig. 7:**
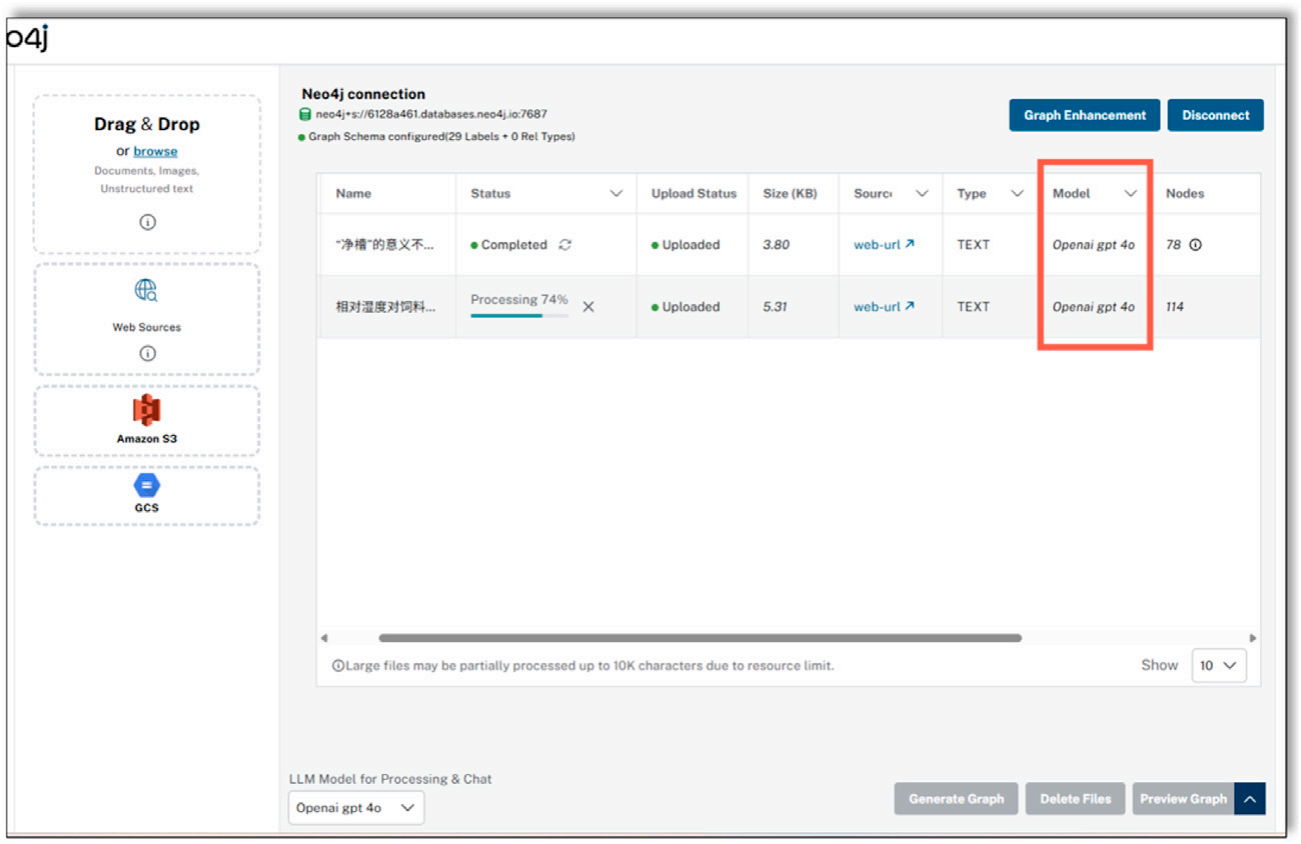
Automatic extraction of large language models.

The llm_graph_builder framework (https://github.com/BinNong/llm-graph-builder) was used to automate the extraction and construction of a knowledge graph for broiler farming (Fig. 7). In addition to structured book content, web-based text was incorporated to broaden the graph’s coverage. Prompt templates were generated from the entity and relation dictionaries to guide GPT-4o in extracting candidate triples. Fig. 6 shows the prompt configuration based on broiler chicken domain knowledge, defining the key entity types and relationship categories used to guide the language model during the triplet extraction process.

**Fig. 8:**
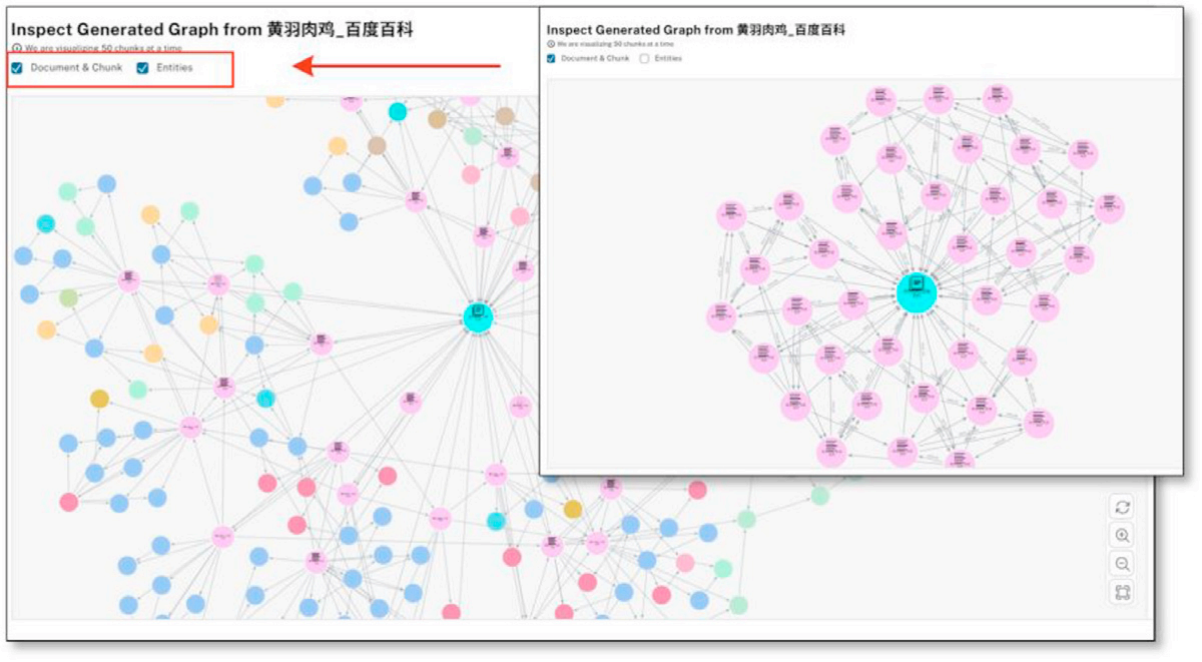
Visualization of corpus chunks and linked entities.

To mitigate hallucination—an inherent challenge in LLM-based triple extraction—we rely on the document-grounded generation mechanism provided by the llm-graph-builder framework. This framework segments the domain corpus into fine-grained text chunks and performs semantic retrieval to fetch relevant contexts for each extraction task. As illustrated in Fig. 8, each document chunk node (blue) is connected to multiple entity nodes (colored by type), clearly showing the traceable path from triples back to their supporting context. This architecture not only prevents hallucinations but also facilitates manual verification and downstream QA integration.

To enhance reliability and domain consistency, an expert-in-the-loop approach was adopted. Domain experts defined rule-based constraints (e.g., “Feeding strategies must correspond to growth stages”) to filter biologically implausible outputs. To achieve a balance between efficiency and reliability, most high-confidence outputs—especially those generated by structured prompts based on book-derived dictionaries—do not require manual revision. Only outputs involving low-frequency entities, ambiguous contexts, and critical domain-sensitive relationships (such as disease diagnosis, breeding standards, and environmental conditions) are subject to manual review.

### 4.3 Knowledge graph construction

**Fig. 9:**
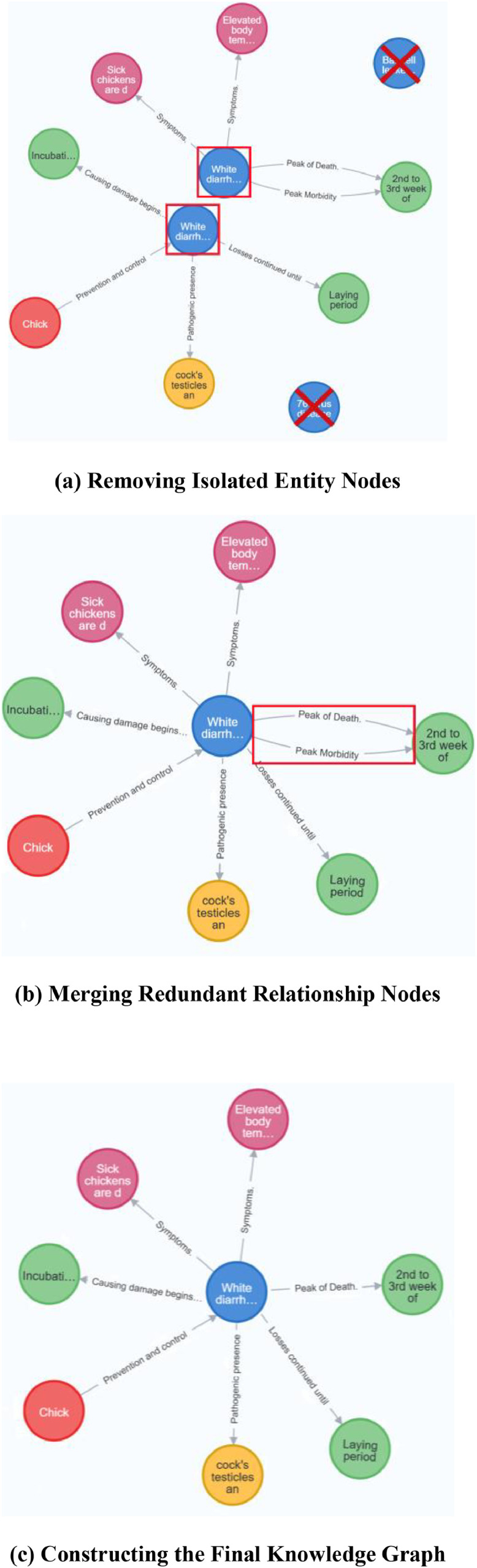
Knowledge integration process.

All extracted triples were organized and cleaned [39]. First, orphaned entities with no relational links were identified and removed, improving the graph’s overall accuracy and coherence. These isolated entities often result from previous errors or incomplete data processing. Their removal ensured a more concise and efficient knowledge graph (Fig. 9).

Second, entity and relation disambiguation was conducted. A semi-automated process combining rule-based matching and expert review was applied. Entries with similar meaning but different expressions were identified and merged to reduce redundancy, improve semantic consistency, and enhance graph interpretability. Specifically, entity disambiguation involved name standardization (e.g., merging “broiler feed additives” and “additives (broiler)”), string similarity metrics (e.g., Levenshtein distance), and context co-occurrence analysis to assess conceptual overlap. Relationship disambiguation was achieved through vocabulary mapping and syntactic analysis to unify similar relationship expressions (e.g., “applicable to” and “corresponding to” were standardized as “compatible”). Entries with low similarity scores (below 0.85) or inconsistent context were reviewed by domain experts, and consensus was reached through discussion. Finally, all processed triples were imported into Neo4j for visualization and graph rendering.

### 4.4 Application

**Fig. 10:**
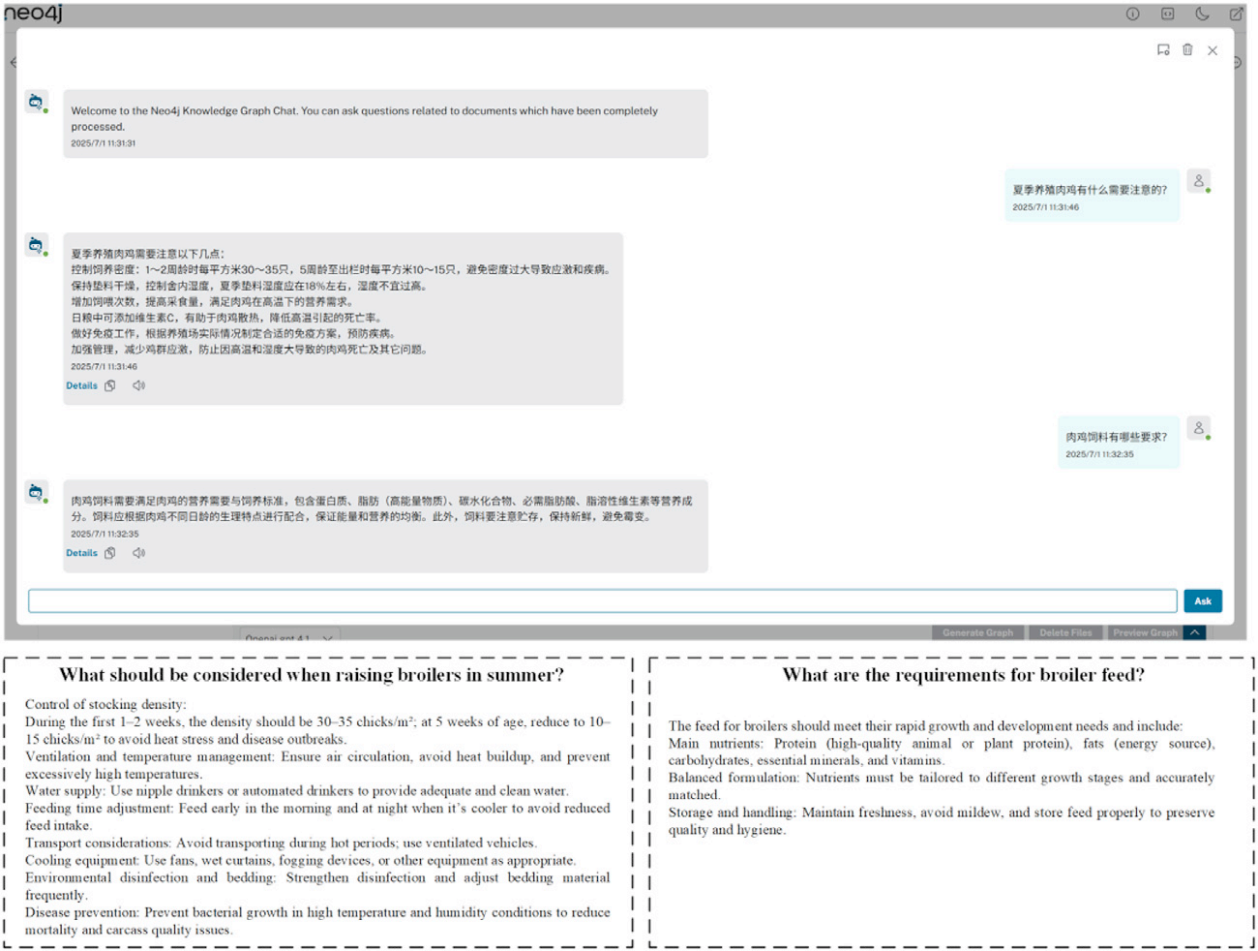
Question-answering (QA) system based on Neo4j.

To showcase the knowledge graph’s potential in downstream applications, a Neo4j-based question-answering (QA) system was developed (see Fig. 10). In this system, users interact with the knowledge graph by submitting natural language queries related to broiler farming. For example, queries like “What factors nsidered when raising broilers in summer?” or “What are the nutritional requirements of broiler chickens?” trigger responses based on the structured triples in the graph. This interactive QA functionality illustrates how knowledge graphs can support intelligent information retrieval and decision-making in poultry production, demonstrating their value beyond static data storage.

**Fig. 11:**
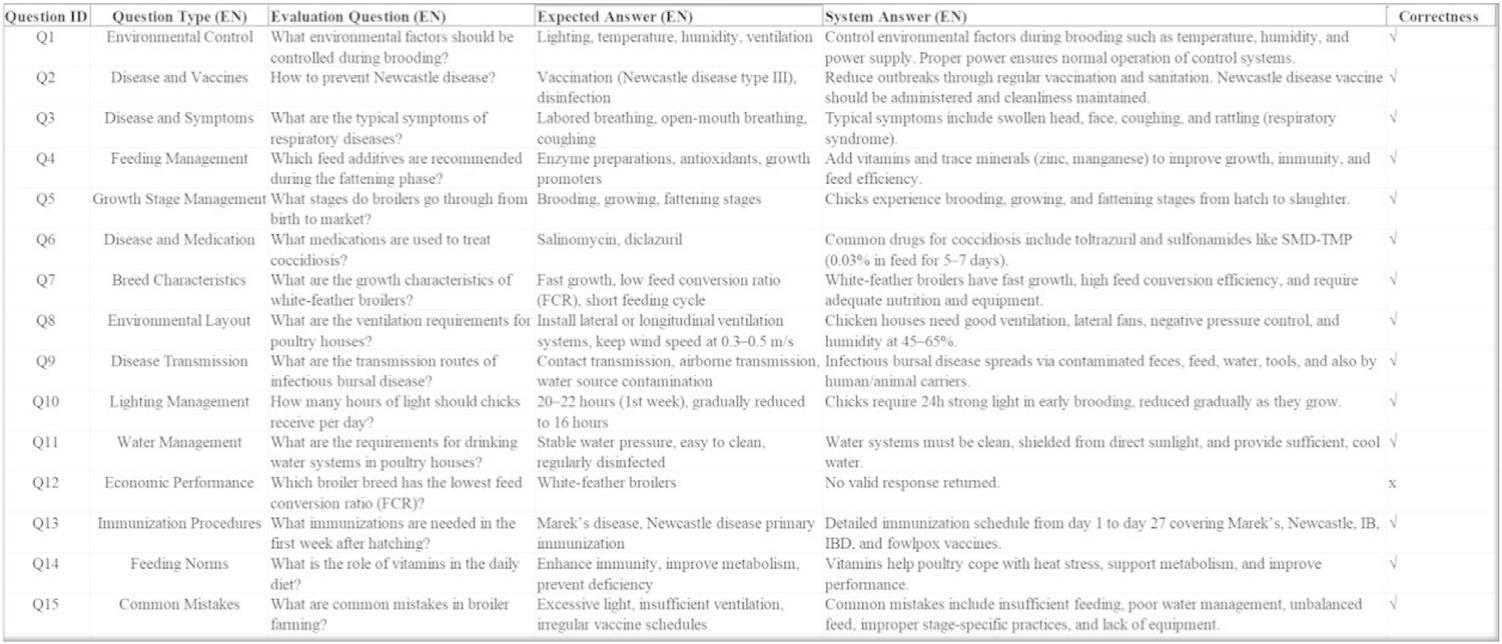
Evaluation of a question answering (QA) system based on Neo4j.

To evaluate the usability of the constructed knowledge graph in downstream applications, we used 15 real-world questions related to broiler farming and evaluated them through a question-answering (QA) system. The QA system implemented based on Neo4j provided accurate and structured answers to 14 of the 15 questions, with an overall accuracy rate of 93 %, as shown in Fig. 11.

## Limitations

While the dataset provides a structured foundation for research and intelligent broiler farming applications, it has notable limitations that require further refinement. Specifically, the current version of BroilerKG is built primarily from textual data. However, real-world broiler farming relies heavily on multimodal information, including images, videos, and sensor data [40]. These data modalities offer critical insights for real-time environmental monitoring, growth assessment, and early disease detection—areas that can be further enhanced through computer vision and IoT technologies.

To overcome this limitation, future work will aim to integrate multimodal data into the knowledge graph. One promising approach is cross-modal entity alignment, where visual or sensor-based observations (e.g., thermal anomalies or lesion images) are linked to corresponding textual entities and relations in the graph. Methods such as CLIP-based image-text embedding, TCN-based signal encoding, and transformer-based multimodal fusion may be used to link heterogeneous data with knowledge triples. This integration would enable the creation of a richer, context-aware, and continuously updatable knowledge graph aligned with the needs of smart poultry farming.

Furthermore, although this study focuses on Chinese-language textual data in the broiler farming domain, the proposed pipeline is modular and theoretically extensible. By adjusting the entity-relation dictionaries and prompt templates, it could be adapted to other agricultural domains such as cattle farming, aquaculture, and crop production. However, this potential has not yet been empirically verified due to the lack of well-annotated domain corpora and the significant manual effort required to construct new knowledge bases for each target domain. Future research will explore these extensions and evaluate the pipeline’s scalability and cross-domain generalizability.

## Ethics Statement

The current work does not involve human subjects, animal experiments, or any data collected from social media platforms.

## Credit author statement

Nan Ma: Conceptualization; Methodology; Software. Fantao Kong: Data curation; Writing - original draft. Jifang Liu: Visualization; Investigation. Chenyang Zhang: Supervision. Chenxv Zhao: Supervision. Shanshan Cao: Software; Validation. Wei Sun: Writing - review & editing.

## Data Availability

Mendeley DataBroiler Breeding Knowledge Graph Dataset (Original data).
